# The state-of-art polyurethane nanoparticles for drug delivery applications

**DOI:** 10.3389/fchem.2024.1378324

**Published:** 2024-02-26

**Authors:** Wencong Song, Saz Muhammad, Shanxing Dang, Xingyan Ou, Xingzi Fang, Yinghe Zhang, Lihe Huang, Bing Guo, XueLian Du

**Affiliations:** ^1^ The Fourth Clinical Medical College of Guangzhou University of Chinese Medicine, Shenzhen, Guangdong, China; ^2^ Shenzhen Traditional Chinese Medicine Hospital, Shenzhen, Guangdong, China; ^3^ School of Science, Shenzhen Key Laboratory of Flexible Printed Electronics Technology, Shenzhen Key Laboratory of Advanced Functional Carbon Materials Research and Comprehensive Application, Harbin Institute of Technology, Shenzhen, China; ^4^ Center for Educational Technology, Yulin Normal University, Yulin, China

**Keywords:** drug delivery, polyurethanes, nanoparticles, targeting, image-guided drug delivery

## Abstract

Nowadays, polyurethanes (PUs) stand out as a promising option for drug delivery owing to their versatile properties. PUs have garnered significant attention in the biomedical sector and are extensively employed in diverse forms, including bulk devices, coatings, particles, and micelles. PUs are crucial in delivering various therapeutic agents such as antibiotics, anti-cancer medications, dermal treatments, and intravaginal rings. Effective drug release management is essential to ensure the intended therapeutic impact of PUs. Commercially available PU-based drug delivery products exemplify the adaptability of PUs in drug delivery, enabling researchers to tailor the polymer properties for specific drug release patterns. This review primarily focuses on the preparation of PU nanoparticles and their physiochemical properties for drug delivery applications, emphasizing how the formation of PUs affects the efficiency of drug delivery systems. Additionally, cutting-edge applications in drug delivery using PU nanoparticle systems, micelles, targeted, activatable, and fluorescence imaging-guided drug delivery applications are explored. Finally, the role of artificial intelligence and machine learning in drug design and delivery is discussed. The review concludes by addressing the challenges and providing perspectives on the future of PUs in drug delivery, aiming to inspire the design of more innovative solutions in this field.

## 1 Introduction

**SCHEME 1:**
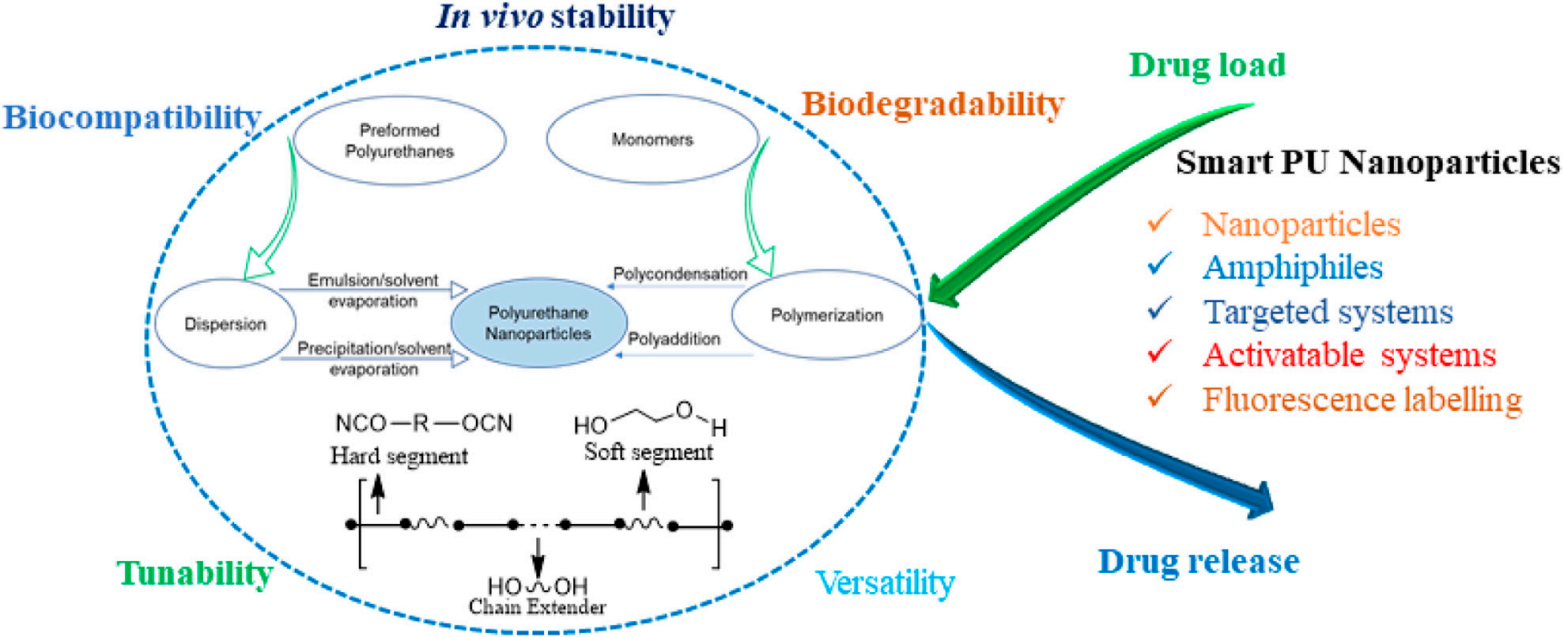
Schematic representation of polyurethane properties and drug delivery application.

Polyurethanes (PUs) are versatile polymers comprising diisocyanates and polyol components and are known for their extinsive applications due to their flexibility, durability, and resilience (Liu H. et al., 2022; Peyrton and Avérous, 2021). PUs can exist in various forms, like flexible foams, rigid insulation, coatings, adhesives, and sealants. PUs boast diverse biomedical uses owing to their adaptable, biocompatible, and resilient nature. Their flexibility makes them ideal for catheters and tubing, lessening the risk of adverse bodily reactions and minimizing tissue damage during insertion (Wendels and Avérous, 2021; Feldman, 2021). Moreover, in crucial medical devices like pacemaker leads and artificial heart components, the durability of PUs and resistance to bodily fluids prevent degradation or adverse responses (Pfensig et al., 2022). PUs films serve as effective wound dressings, creating a barrier against bacteria while enabling oxygen and moisture passage, fostering an optimal environment for wound healing (Morales-González et al., 2022). Their customizability aids tissue engineering by mimicking natural tissue mechanics, supporting cell growth and regeneration within scaffolds (Naureen et al., 2021; Pedersen et al., 2022). Additionally, their lightweight quality, durability, and moldability provide comfort and functionality in prosthetic limbs, braces, and orthotic devices (Naureen et al., 2021). The capacity of PUs to encapsulate drugs and offer gradual release in drug delivery systems, as seen in controlled-release matrices or implant coatings, further demonstrates their versatility **(** Scheme 1 **)**. (Laurano et al., 2021)

PUs have been utilized in various pharmaceutical formulations as more and more monomers became available. Recent literature and reviews mainly focus on the potential of PUs in drug delivery systems with emphasis on general properties and drug administration studies (Gupta, 2023; Nadaf et al., 2023). The drug delivery systems based on PUs exhibit noteworthy promise owing to their straightforward preparation, elevated pharmacological effectiveness, minimal toxicity, and precise control over drug release. Their responsiveness to stimuli, stability over time, and ability to solubilize hydrophobic drugs offer encouraging opportunities for advancing effective therapies within the realm of pharmaceuticals. Here we review the role of segmental structures and properties modulations in designing state-of-the-art polyurethanes for drug delivery systems (DDSs).

**FIGURE 1:**
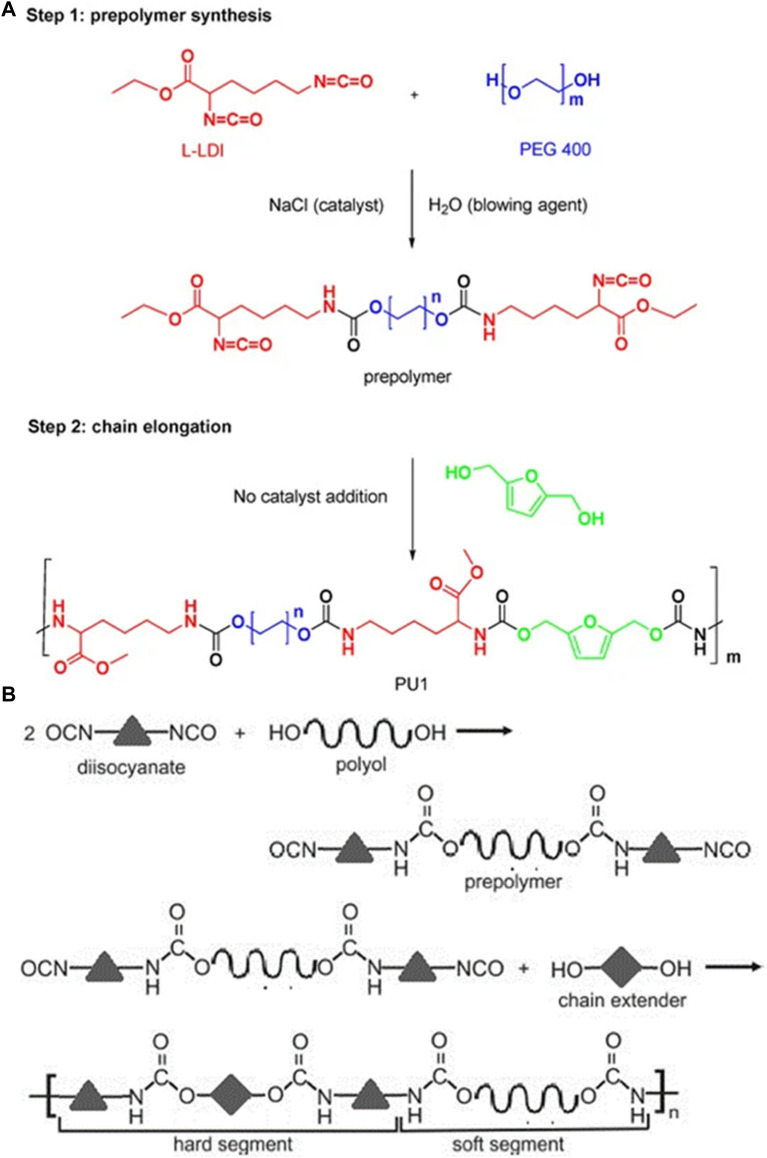
**(A)** Synthesis of biocompatible poly (urethane) from PEG 400 and l-Lysine (Olivito et al., 2023). Reused under Creative Commons Attribution License **(B)** Synthesis of a poly (urethane) network general representation (Szczepańczyk et al., 2021). Reused under Creative Commons Attribution License.

Drug delivery systems (DDSs) are natural, synthetic, or semi-synthetic materials that intermingle with living systems to cope with direct medical treatment and diagnostics (Abbasnezhad et al., 2021). The materials may be biodegradable, bioresorbable, biocompatible, and hemocompatible to perform their function according to the response of the host (Zhang S. et al., 2021). These materials can be divided into composites, polymers, carbon materials, and ceramics. Polyurethanes are one of the most promising and versatile materials, having a segmental block structure with fascinating features like tunable biological, mechanical, physiochemical, and tissue-compatible properties (Yang et al., 2021). The high elasticity and low glass transition temperature (Tg) due to soft segmental structures and high strength, Tg, and melting temperature (Tm) due to hard segments of PUs are unique physiochemical properties in the context of DDSs (Figure 1). (Shoaib et al., 2017a; De Souza et al., 2021)

PUs, due to their adaptable properties and widespread application in medicine, stand out as excellent options for delivering drugs. Their versatility has sparked considerable interest in the biomedical sector, where these have been extensively used in various forms—such as bulk devices, coatings, particles, and micelles—for drug delivery purposes. PUs have found roles in delivering antibiotics, anti-cancer medications, drug-eluting stents, dermal treatments, and intravaginal rings. To ensure the intended therapeutic impact, it is crucial to manage how PUs release drugs, controlling both the pace and duration. (Ghasemi and Ghezelsofloo). There are already commercially available PU-conjugated DDSs, like the Ornibel intravaginal ring, showcasing the adaptability of PU formation, allowing scientists to adjust the properties of the polymer to attain specific drug release patterns (Rajan et al., 2017). PU nanoparticles are the most promising DDS among all the other PU-related DDSs because of greater surface area, high drug distribution, enhanced selectivity, and increased drug-delivering efficacy. PU nanoparticle DDSs are extensively employed as the most efficient drug delivery carriers for stimuli-responsive drug delivery. In this review, the preparation and the physiochemical properties of PU nanoparticles for the use of drug delivery will be discussed. We will also discuss how the efficiency of DDSs depends on the structure of PU nanoparticles. This review also demonstrates the use of state-of-the-art PU nanoparticles in DDSs. The use of developed PU nanoparticle DDSs to treat various diseases will also be discussed.

## 2 Preparation of polyurethane nanoparticles

Polymeric nanoparticles have become a potent tool for delivering drugs and diagnosis in the past few decades (Mishra et al., 2013). Typically characterized as compact colloidal substances consisting of macromolecular or molecular assemblies spanning a diameter of 1–500 nm, nanoparticles possess unique features such as elevated kinetic stability, diverse structural variations, rigid morphology, the capacity to integrate a variety of hydrophilic or hydrophobic medications, and numerous possibilities for functionalization (Rao, 2011; Morral-Ruíz and Melgar-Lesmes, 2016). Polymeric nanoparticles show potential as a therapeutic approach in the creation of nanostructured materials for drug delivery due to the mentioned characteristics. In this context, the crucial steps involve the careful selection of suitable polymers and their proper formulation to achieve the desired properties. Polyurethanes are particularly captivating in this regard, given their noteworthy attributes, including robust mechanical strength, biodegradability, biocompatibility, and versatile synthetic capabilities (Morral-Ruíz et al., 2016).

Polymeric nanoparticles can be easily crafted either by utilizing polymers which are preformed or through the polymerization of monomers directly by employing conventional polymerization or multiple reactions. Techniques like evaporation of the solvent, dialysis, salting-out, and supercritical fluid technology, which involves the rapid increase in the volume of a supercritical solution or its quick conversion into a liquid solvent, can be employed for preparing polymeric nanoparticles from already synthesized polymers. PU nanoparticles are conventionally synthesized from diisocyanates and diols/polyols or diamines through polycondensation or polyaddition reactions. PU nanoparticles can also be prepared directly by polymerization of monomers by using different emulsion techniques such as mini-emulsion, micro-emulsion, surfactant-free emulsion, and interfacial polymerization. These methods facilitate the integration of cell-penetrating molecules, stimuli-responsive linkages, and targeting ligands into the core PU structure. Additionally, they enable the design of nanoassemblies as nanovectors for drug delivery applications (Xu et al., 2010; Wang et al., 2013a).

**SCHEME 2:**
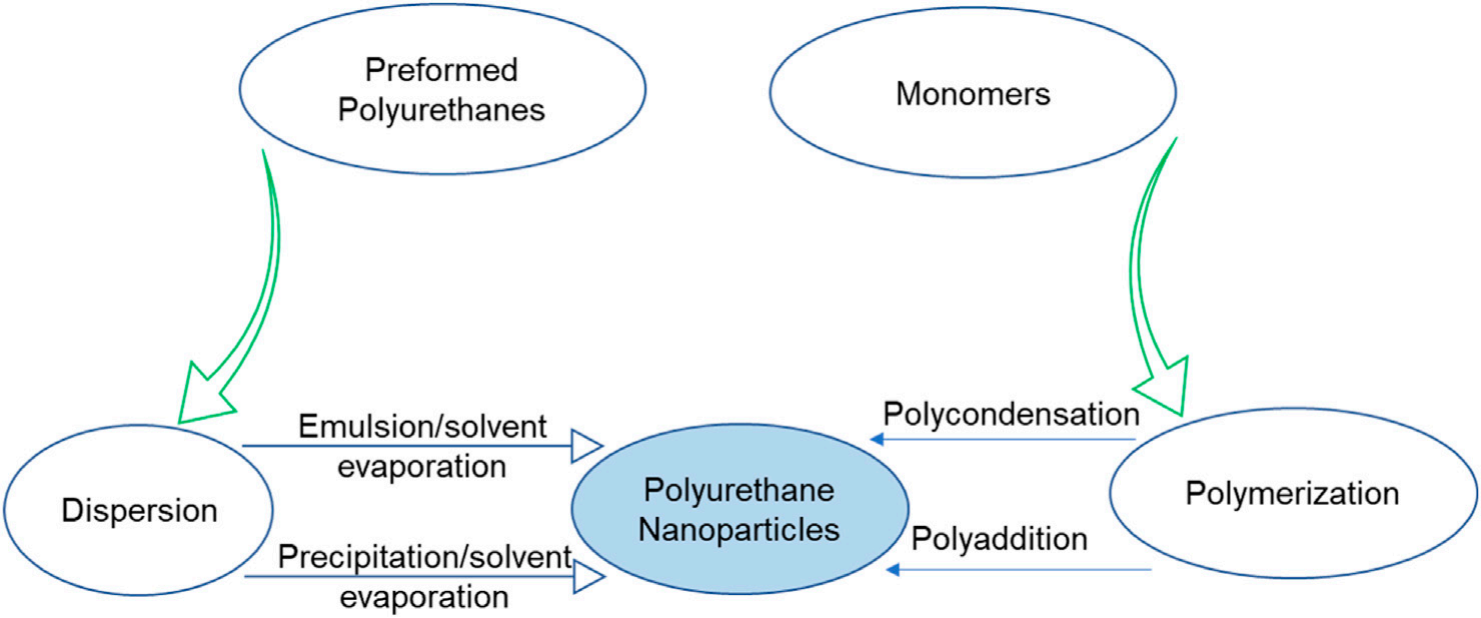
Schematic demonstration of various techniques for preparing of PU nanoparticles.

In addition to the traditional polycondensation or polyaddition reactions involving diisocyanates and diols/polyols or diamines, PUs can engage in reactions with preformed synthetic polymers like poly ε-caprolactone or polysaccharides. This approach aims to achieve polymers that are more biocompatible and biodegradable. Conversely, interfacial polymerization at the interface of two immiscible liquids results in the creation of functionalized structures. These structures exhibit multimodal distributions with varying sizes and the coexistence of monomers, empty micelles, microemulsion droplets, and nanoemulsions. The microemulsion droplets and nanoemulsions serve as templates and micro-nano-reactors for the synthesis of PU nanoparticles (Borcan et al., 2023). Several techniques such as emulsion/solvent evaporation and nanoprecipitation/solvent evaporation have been employed for the formation of PU nanoparticles from already synthesized polymers. The synthesis of PU nanoparticles from polymerization and prepolymers is demonstrated in Scheme 2. Additionally, desolvation, dialysis, ionic gelation, nanoprecipitation, solvent evaporation, salting out, spray drying and supercritical fluid methods are utilized for polymeric nanoparticles synthesis. However, the choice of an appropriate method depends upon various factors.

### 2.1 Amphiphilic polyurethane nanoparticles

Molecules possessing both polar and nonpolar components, known as amphiphilic molecules, organize at boundaries and interact to form clusters in a system (Kim et al., 2004). Polymer surfactants, such as polyethylene glycol (PEG) polymers like Pluronics, have been extensively explored for various applications in aqueous environments. These applications encompass tasks like crystallization, modifying surfaces for biocompatibility, regulating nanoparticle aggregation in solutions, and facilitating drug delivery. The different stimuli responsiveness of anionic and cationic nanomicelles enrich the library of amphiphilic molecules for drug delivery applications. The neutral surfactants or block copolymers forming micelles or aggregates respectively, can control the formation of different drug carrier architectures. Pluronics, a non-ionic block copolymer, when present at low polymer concentrations, exist as separated polymer coils or monomers, though in highly dilute solutions. However, as the polymer concentration or temperature rises, the initiation of micelle formation occurs, reaching the critical aggregate concentration or critical micelle concentration. Recently, the main interest of scientists in active materials has shifted towards designing polymer nanoparticles with intelligent, or smart, behavior. Adaptable polymer nanoparticles respond to their surroundings by controlling the movement of ionic and molecular species, altering hydrophilicity, and influencing the bond of various species when exposed to external stimuli (Aghaghafari and Zamanloo, 2019). Depending on the characteristics of the solvent environment, polymers featuring hydrophobic and hydrophilic head groups can create micelle and inverted micellar assemblies. These self-assembled nanoparticles exhibit size and morphology control, functioning effectively in nonpolar and polar environments and offering ability for diverse applications. The extensive variety of active synthetic copolymers presents chances to design a wide array of smart materials with variations in size, chemical structure, and chain conformation (Kim and Shim, 2004; Tsai et al., 2015).

**FIGURE 2:**
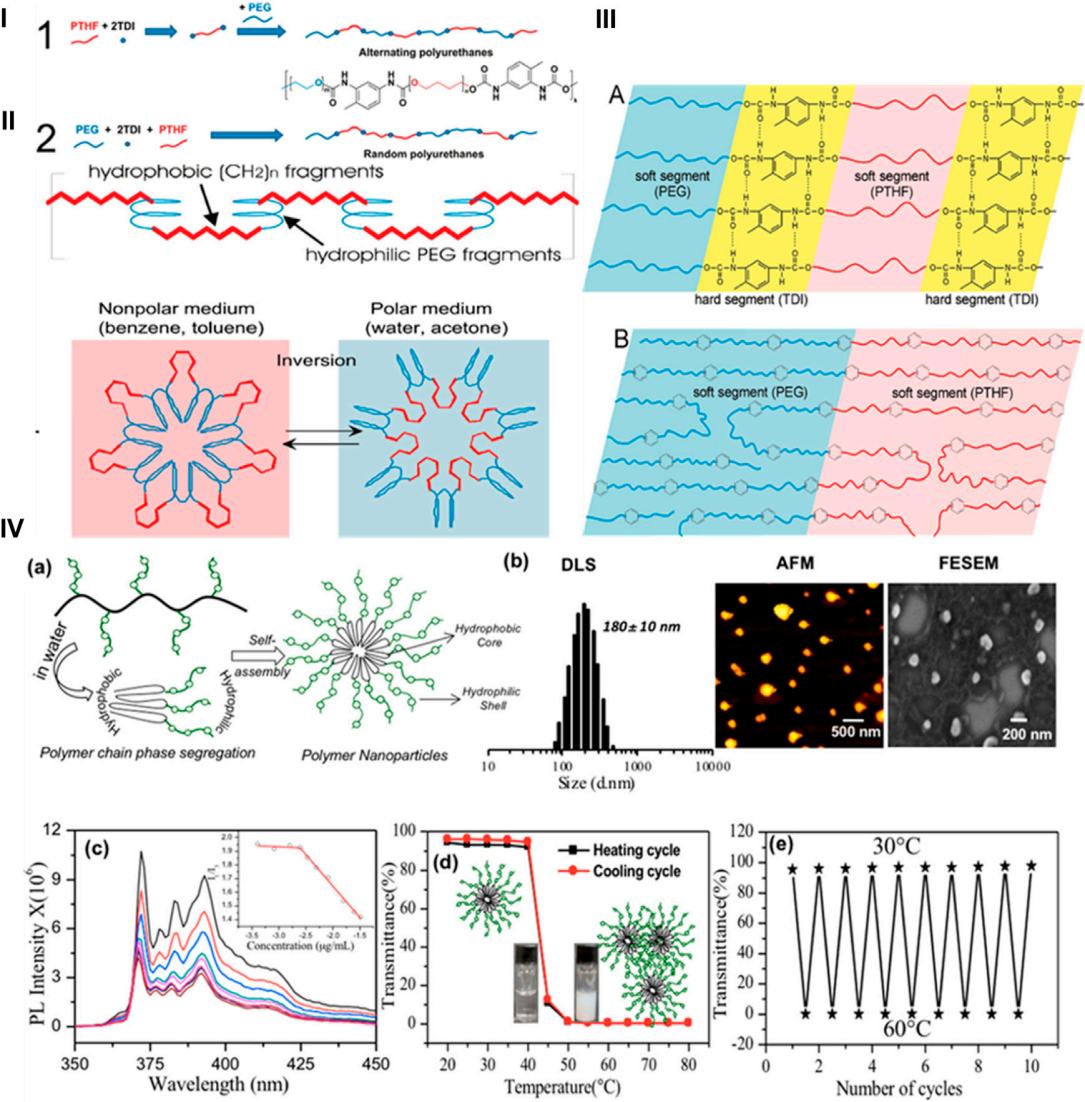
**(i)** Scheme and synthesis of amphiphilic PUs. **(ii)** Micellar and invertible micellar representation in polar and non-polar media. **(iii)** Microphase separation in alternate polymer **(A)** and copolymer **(B)** (Hevus et al., 2010). Reproduced with permission from ref. no. 33. Copyrights 2010, AMERICAN CHEMICAL SOCIETY. **(iv) (A)** Illustration of the self-assembled polar polyurethane forming nanoparticles. **(B)** Histogram from DLS, AFM image, and FE-SEM image of polymeric nanoparticles. **(C)** Determination of the critical micelles concentration of polymeric nanoparticles using the pyrene probe in aqueous medium. **(D)** Graph depicting the percentage transmittance to showcase the thermoresponsive behavior of polymeric nanoparticles in aqueous medium during heating and cooling processes. **(E)** Graph illustrating the variation in %T of polymeric nanoparticles over 10 consecutive heating and cooling sets at 30°C–60°C. The polymer amount was preserved at 1.0 mgmL^-1^ for %T measurements (Joshi et al., 2019). Reproduced with permission from ref. no. 34. Copyrights 2019, AMERICAN CHEMICAL SOCIETY.

PU nanoparticles containing alternate polar and nonpolar segments have illustrated reduced surface energies when bare to water and air, suggesting the potential for switchable behavior. The correlation between the properties of the surfaces of copolymers having both nonpolar and polar components and the formate of PUs with polyol soft sections has been explored. For instance, Hevus et al. synthesized innovative amphiphilic invertible polymer nanoparticles using PEG as the polar element and polytetrahydrofuran (PTHF) as the nonpolar section of the polymer. PEG is soluble in both aqueous and nonaqueous medium, while PTHF is not soluble in aqueous media. Two conformations have been developed for polar polyurethane nanoparticles: one with alternate distribution (PEG-alt-PTHF) and another with arbitrary distribution (PEG-co-PTHF) of polar and nonpolar sections (Figures 2i–2iii). They hypothesized that the alternating arrangement facilitates quick swapping of the polymer structure in response to changes in polarity of the surroundings. On the other hand, the arbitrary circulation provides polymer nanoparticles with variable size of nonpolar and polar segments, leading to diverse capabilities such as micelle formation and inversion (Hevus et al., 2010).

Similarly, Dheeraj et al. utilized a solvent-free melt transurethane polycondensation method to synthesize PU nanoparticles based on l-lysine. Customized polyfunctional l-lysine monomer units were created by changing NH_2_ groups into urethanes, while simultaneously covering the carboxylic groups as amide lockets. These l-lysine monomer units projected to melt transurethane polymerization to yield heavy straight chain polyurethanes. Additionally, a novel polar l-lysine monomer unit featuring a PEG-350 chain locket was designed. On polymerization, this monomer produced distinct polar aliphatic PUs (APUs). The APUs underwent core-shell self-assembly in water, forming nanoparticles with a size of less than 175 nm and displaying remarkable encapsulation capabilities. The APU nanocarriers exhibited thermoresponsive behavior, transitioning from a solution to suspension above the lower critical solution temperature (LCST) at 41°C–43°C, equivalent to the temperature of cancerous tissues (Figure 2iv) (Joshi et al., 2019). The aforementioned research affirms that aliphatic polyurethanes and their polar nanocarriers represent highly promising options for biomedical applications. PUs from these categories, produced through inverse micellization and solvent-free melt transurethane processes, have significant potential for applications requiring stimuli responsiveness.

### 2.2 PU nanoparticles formulation

Achieving the clinical application of nanoparticle therapeutics poses numerous challenges. Firstly, there is a need for innovative drug delivery vectors that exhibit both high delivery efficiency and low cytotoxicity across diverse cell types. Secondly, drug molecules should be designed to enhance their pharmaceutical properties. Thirdly, the nanoparticle-drug formulations must align with their intended therapeutic applications. Lastly, the tunability of nanoparticle formulations is crucial to broaden the chemical space of their applications. Overall, nanoparticle formulations have significantly contributed to public health during the COVID-19 pandemic. To expand their clinical utility, interdisciplinary research efforts from fields such as chemistry, engineering, materials science, pharmaceutical sciences, and medicine must be integrated (Wang et al., 2021).

A key challenge in the development of drug-delivery nanoparticles is selecting the appropriate polymer, as it determines the final properties of the colloidal system and, consequently, the administration route. Various biodegradable polymers, particularly those from the polyester family such as poly (ε-caprolactone) (PCL), poly (D, L-lactide) (PLA), poly (D, L-lactide-co-glycolide) (PLGA), and their copolymers, have been employed as matrix materials for drug delivery due to their tendency to be reabsorbed by the body without any side effects. Different methods have been utilized for nanoparticle formulations, with solvent displacement being extensively employed in the formation of nanoparticles using various biodegradable polyesters for drug release. Mattu et al. introduced biodegradable polyesterurethanes (PUR) based on PCL blocks as new materials for nanoparticle synthesis through solvent displacement. These PUR nanoparticles show a slightly faster decomposition rate, significantly greater encapsulation ability, a larger, and more precise release ability (Mattu et al., 2012; Politecnico et al., 2012).

Zhang, Haolan, *et al.* integrated polythioketal polyurethane nanoparticles with dexamethasone (PTKU@DEX NPs) as a nanocarrier responsive to reactive oxygen species (ROS) for the treatment of osteoarthritis *in vivo*. These PTKU@DEX NPs effectively scavenged various types of ROS, leading to polymer degradation. The ROS-responsive drug delivery and scavenging capabilities of PTKU showed notable therapeutic outcomes, approaching those of normal cartilage, in the *in vivo* treatment of osteoarthritis (Zhang H. et al., 2021). In a separate investigation, Roshni Iyer and colleagues developed polyurethane nanoparticles (GPUs) that are responsive to glutathione (GSH). These nanoparticles utilized a polyurethane with a GSH-cleavable disulfide bond, allowing them to react to increased GSH levels within lung cancer cells. The fabrication of these polyurethane nanoparticles involved a single emulsion and a mixed organic solvent method. The resulting GSH-sensitive nanoparticles loaded with cisplatin (CGPU) exhibited a release of cisplatin that depended on the dose of GSH (Iyer et al., 2020a). These different formulations of PU nanoparticles have the ability to be used for drug delivery on demand to overcome different medical complications.

## 3 Polyurethane nanoparticles for drug delivery

The efficiency of the DDSs can be significantly enhanced by employing them in the form of nanoparticle systems because of their better water solubility, drug distribution, biocompatibility, and targeting ability (Pabari et al., 2021). Polyurethane nanoparticles are tiny particles made of polyurethane employed in DDSs due to their unique properties that make them suitable for transporting drugs to specific bodily targets. PU nanoparticles have the ability to encapsulate drugs within their structure. This encapsulation protects the drug from degradation and ensures controlled release at the targeted site. The engineered nanoparticles can be specific in targeting body cells or tissues. Functionalization with ligands or antibodies allows them to recognize and bind to specific receptors on cell surfaces, enhancing their specificity (Omrani et al., 2017; Shoaib et al., 2018). The production of PUs involve a wide variety of materials including the use of a monomer, or a low-molecular-weight pre-polymer solvent. The creation of the carbamate (urethane) linkage is a representation of the outcomes of the primary processes in PU synthesis. Their properties are dependent on the incorporation of different groups like ester, ether, urea, aromatic, carbodiimides in the polymeric structures. The molecular structure, rigidity, and flexibility control the overall PU systems and their applications.

Moreover, their small size (usually 10–100 nm) enables easy transport through biological barriers and enhances their blood circulation time. Additionally, their stability helps in preserving the integrity of the drug until it reaches the desired location. PU nanoparticles offer controlled and sustained release of drugs. By modifying the structure of nanoparticles, the rate of drug release can be tailored to ensure a prolonged therapeutic effect and reduce the frequency of drug administration. PUs are often biocompatible which can be well-tolerated by the body, reducing the risk of adverse reactions when used as a drug carrier. Overall, PU nanoparticles offer a promising avenue for improving DDSs by enhancing drug stability, targeting specific cells or tissues, and providing controlled release kinetics, ultimately increasing the therapeutic effectiveness of various medications (Wang et al., 2013b).

For example, PUs designed for complete degradation triggered by intracellular GSH and acidic environments have been overlooked in targeted controlled drug delivery. To address this, novel waterborne polyurethane (WPU) nanoparticles capable of breaking down in response to GSH and acid were explicitly developed for delivering the lipophilic anti-cancer drug doxorubicin (DOX) (Iyer et al., 2020b). These resulting PUs spontaneously form nanoparticles in water, as observed through DLS and SEM, demonstrating rapid inflammation and disintegration in an environment mimicking intracellular reduction and acidity. *In vitro* release experiments revealed that in 10 mM GSH at pH 6, 74% of the loaded with DOX was released from these WPU nanoparticles within 24 h. MTT assay results indicated that the cytotoxicity of DOX-loaded WPU nanoparticles against A375 human malignant melanoma cells was comparable to free DOX, while blank WPU nanoparticles exhibited low cytotoxicity. These findings highlight the potential of GSH and acid-responsive WPU nanoparticles as effective carriers for intracellular drug delivery, responsive to both pH levels and redox conditions. Moreover, the PU DDSs can also act as activatable DDSs when coated with some targeting agents according to the requirement. The activatable PU DDSs can be the most advanced delivery system in treating various diseases, including cancer (Zanetti-Ramos et al., 2009; Wang et al., 2013b).

**FIGURE 3:**
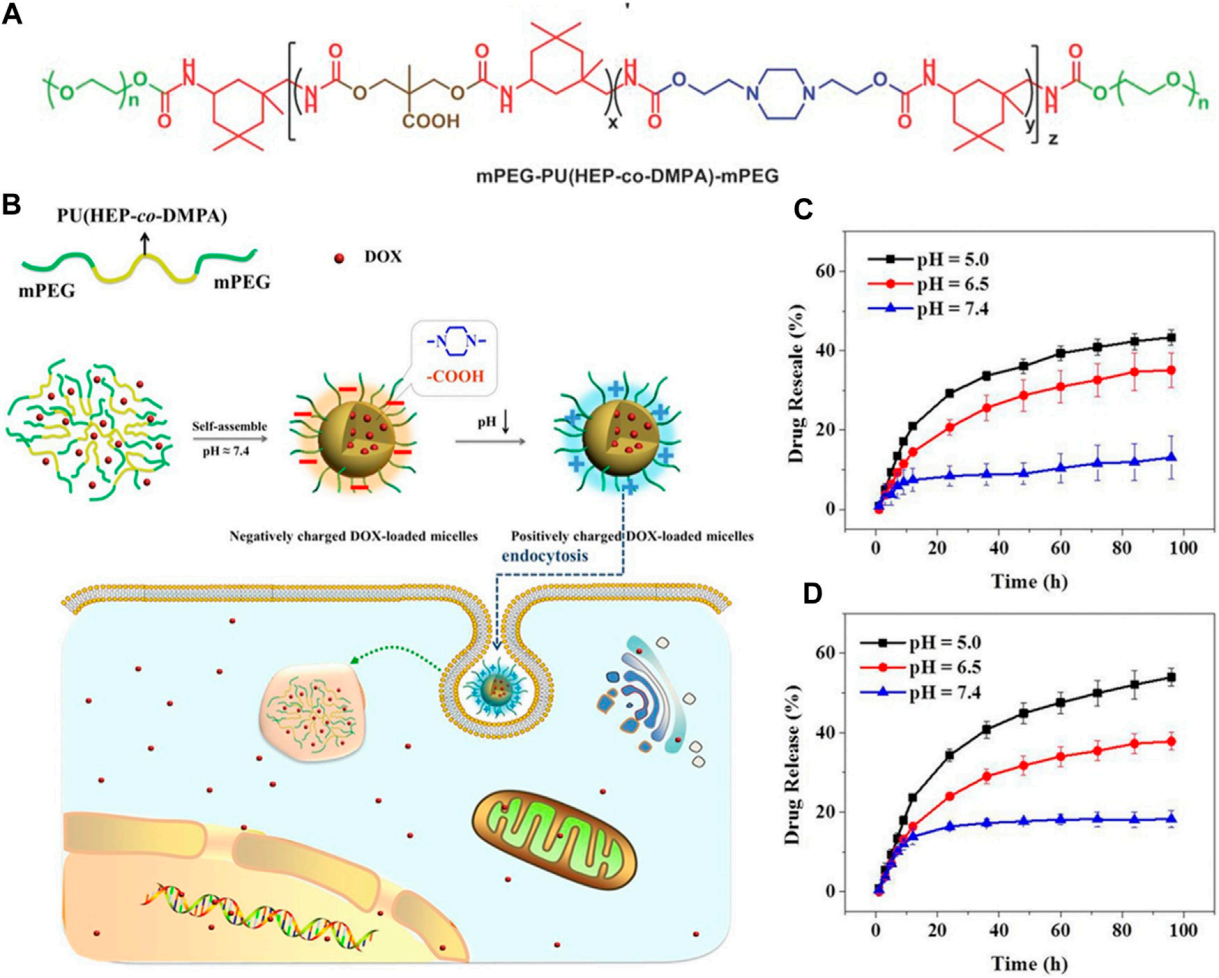
**(A, B)** Reactions and scheme of pH responsive PUs containing carboxylic acid groups, methoxyl-poly (ethylene glycol) (mPEG), and piperazine groups as charge reversal intelligent intracellular drug delivery carriers. **(C , D)** Polystyrene-polyurethanes of different compositions loaded with the drug, PS-PU2-DOX, and PS-PU3-DOX under different pH conditions respectively, at 37°C (He et al., 2016). Reproduced with permission from ref. no. 47. Copyrights 2016, WILEY.

Polymeric nanomicelles DDSs with acid-sensitive charge-reversal properties in response to the acidic tumor microenvironment, facilitating their entry into cancer cells have been the focus of many studies. For instance, Liu et al. utilized carboxyl groups and tertiary amine groups to synthesize amphiphilic PUs containing carboxylic groups (PUC) and amine groups (PUN). The stability and cellular studies conducted indicated that PUC-PUN micelles displayed remarkable stability under simulated normal physiological conditions and significantly enhanced cellular uptake efficiency. These micelles exhibited minimal cytotoxicity against SGC-7901 and MGC-803 cells, while PUC-PUN/DOX micelles showed increased cytotoxicity compared to both pure DOX and PUN/DOX micelles. Moreover, *in vivo* experiments assessing antitumor activity revealed that PUC-PUN/DOX micelles demonstrated superior tumor inhibition efficacy and safety when compared to pure DOX. The study highlights that charge reversal is a potential strategy for designing efficient DDSs (Liu J. et al., 2022). He, Wanying, et al. prepared pH responsive PUs containing methoxyl-poly (ethylene glycol) (mPEG), carboxylic acid groups, and piperazine groups as charge reversal intelligent intracellular drug delivery carriers. They found that the charge-reversal property of the prepared PU can improve the cellular uptake behavior and intra-cellular drug release in both HeLa cells and MCF-7 cells (Figure 3). (He et al., 2016)

**FIGURE 4:**
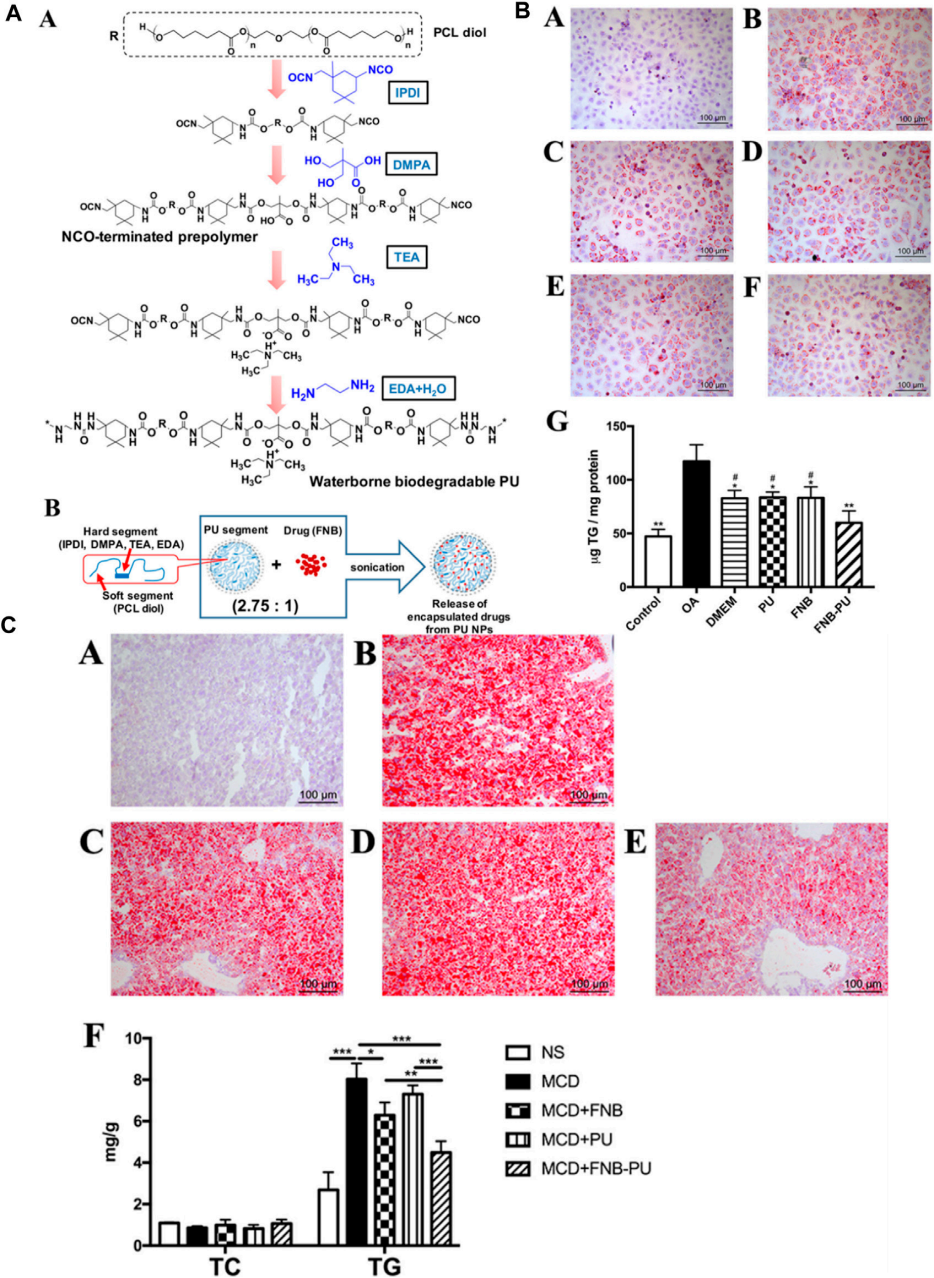
**(A)** Polymer and polymer loaded with drug structures. **(A)** Formation and molecular composition of polymer. **(B)** Diagrammatic representation of the nanoparticle form of polymer loaded with drug. **(B)** Staining **(A–F)** and TG Content **(G)** in specimen Cells during the *in vitro* disease treatment experiment. **(C)** Staining of Liver **(A–E)** and Lipid Content **(F)** in Mice (Cao et al., 2018). Reproduced with permission from ref. no. 48. Copyrights 2018, AMERICAN CHEMICAL SOCIETY.

Furthermore, Cao et al. synthesized PU-based nanoparticles loaded with a drug known as fenofibrate (FNB-PU) to demonstrate the *in vitro* and *in vivo* efficacy of the drug for the treatment of nonalcoholic fatty liver disease. They prepared PU nanoparticles by a green process, and FNB was encapsulated into the PU nanoparticles by a facile sonication method. The low molecular weight of the single chain which is about 197 kDa and the weight of each nanoparticle determined by single-angle scattering data was approximately in the range of 6 - 8 × 10 ^6^. It means that each nanoparticle contained 300–400 molecules having a tight and compact structure with 39 nm in size. When loaded with the drug, the size of the FNB-PU was about 57.7 nm. The enlargement in the size of PU nanoparticles demonstrated successful encapsulation of FNB with PU nanoparticles. The FNB-PU displayed two modes of drug release in the *in vitro* system, initial burst of drug release and subsequent slower drug release. The encapsulation of FNB into the PU nanoparticles increased the drug release by about 2.8 folds to the FNB aqueous suspension. Finally, the FNB-PU was orally administrated to a mouse model suffering from nonalcoholic fatty liver to check the *in vivo* efficacy of the drug release. The *in vivo* results demonstrated 8.87 folds increase in the drug release in the case of FNB-PU as compared to crude FNB. The enhancement in the drug release mentioned in the literature was because of the endocytosis of anionic PU micelles (Figure 4). (Cao et al., 2018)

## 4 Physiochemical properties of polyurethanes (PUs) and drug release

Essentially, understanding how drugs are released from polymers often involves analyzing the process through a mass transport perspective, commonly employing Fick’s law of diffusion for modeling. Typically, the drug is either dispersed throughout the polymer structure or encapsulated within it. For systems where the drug is delivered as an encapsulated payload or at concentrations below a critical level, various mass transport phenomena govern the delivery process: the drug must 1) dissolve into the polymer; 2) diffuse through it; 3) pass through a layer of stagnant liquid; and 4) convective transfer into the surrounding environment. When the drug concentration is exceeded, it still proceeds via these transport pathways but can now diffuse through channels that are plentiful in the drug in the material. Moreover, the polymer characteristics, including swelling, erosion, and degradation rate, can affect how quickly drugs are delivered. Additionally, different covalent and non-covalent interactions are involved in drug formulations and release. The way that pharmaceuticals are released over time can also be changed by more advanced methods including covalently attaching the drug to the polymer, using materials that respond to stimuli, or adding pore formers into the formulation (Allami et al., 2021).

### 4.1 Pairing of polymer and drug

A primary method for regulating drug release in PU systems is carefully pairing the polymer and drug. This choice significantly affects two crucial factors governing mass transfer: 1) how well the drug dissolves in the polymer and 2) how easily the drug moves through the polymer. In systems where the drug is either encapsulated or dispersed below a specific concentration, low solubility within the polymer can lead to slower delivery rates due to minimal differences in drug concentration between the polymer and its surroundings (Barman et al., 2023). Conversely, higher drug solubility can accelerate release by creating a more significant concentration disparity between the PUs and the environment. Similarly, the movement of the drug through the polymer affects delivery speed; higher diffusivity leads to faster release compared to lower diffusivity systems (Yu et al., 2014). The chemical structure of the PUs strongly influences drug solubility and diffusivity within the polymer. Factors like PU crystallinity, molecular segment weight, and the balance between hard and soft segments determine how well the drug dissolves and moves through the PU matrix. Moreover, by controlling the PU crystallinity, molecular weight, and balance between hard and soft segments, PU DDSs can be designed according to the respective disorder in the biological system (Mahanta et al., 2015).

### 4.2 Dispersed drug delivery approach

An alternative method for impacting the release of drugs from polyurethanes (PUs) is to decide between utilizing a payload or dispersed drug delivery strategy. Initially, contemplate drug delivery using a payload across a PU membrane, presuming a flat system with limited polymer swelling or breakdown during drug release. In this scenario, numerous elements influence drug delivery, including drug concentrations in the payload, polymer, and surroundings; their proximity to saturation points; concentration gradients within the system; drug diffusivity; and the length of the diffusion path (Shoaib et al., 2017b; Kandavilli et al.; Li et al., 2023). Specifically, the drug concentration in the payload can alter the rate equation governing drug release. If the concentration of payload of the drug surpasses the solubility limit of the polymer, new drug molecules can replace those eluting into the environment, leading to a constant concentration gradient within the polymer and zero-order diffusion kinetics (Akduman et al., 2016).

**FIGURE 5:**
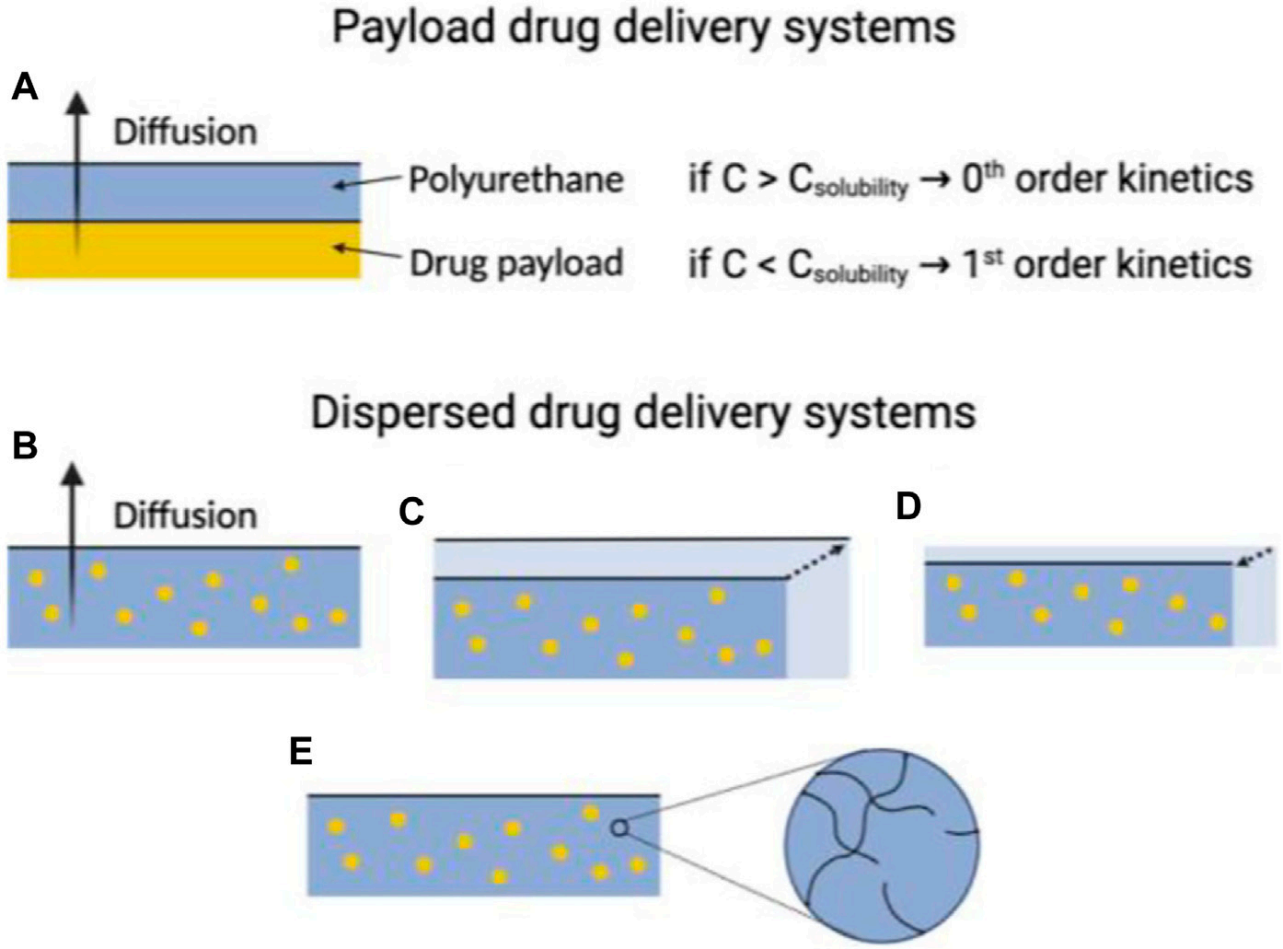
The utility of PUs in drug delivery systems is influenced by their physiochemical characteristics. In instances where PUs used in payload delivery systems exhibit no swelling or degradation, the drug delivery rate is primarily determined by the amount of the payload of the drug relative to its limit of solubility within the polymer **(A)**. For dispersed drug delivery systems, the drug delivery rate can significantly vary, depending on the physiochemical properties of the polyurethane. These properties include whether the macromolecule is **(B)** non-degradable and non-swelling, **(C)** swells when exposed to aqueous environments, **(D)** undergoes erosion, or **(E)** degrades within a time frame comparable to the drug delivery window.

On the flip side, drug release frequently adheres to first-order kinetics when the drug payload concentration remains under the solubility threshold. Another approach involves dispersing the drug within the polymer matrix. In the case of non-swelling and non-degrading polyurethanes (PUs), both the drug loading and device structure play a role in determining drug release kinetics (Figure 5). When the drug concentration stays below the solubility limit, the rate of drug release is commonly represented by an exponential function of time. In contrast, excess drug situations often follow the Higuchi equation, where the released drug fraction correlates with the square root of time. However, the adjustment of the drug payload and its solubility can be controlled by considering the structure of the polymer and the microenvironment of the biological medium under consideration (Virlley et al., 2023).

### 4.3 Customizing the physiochemical characteristics of PUs

**FIGURE 6:**
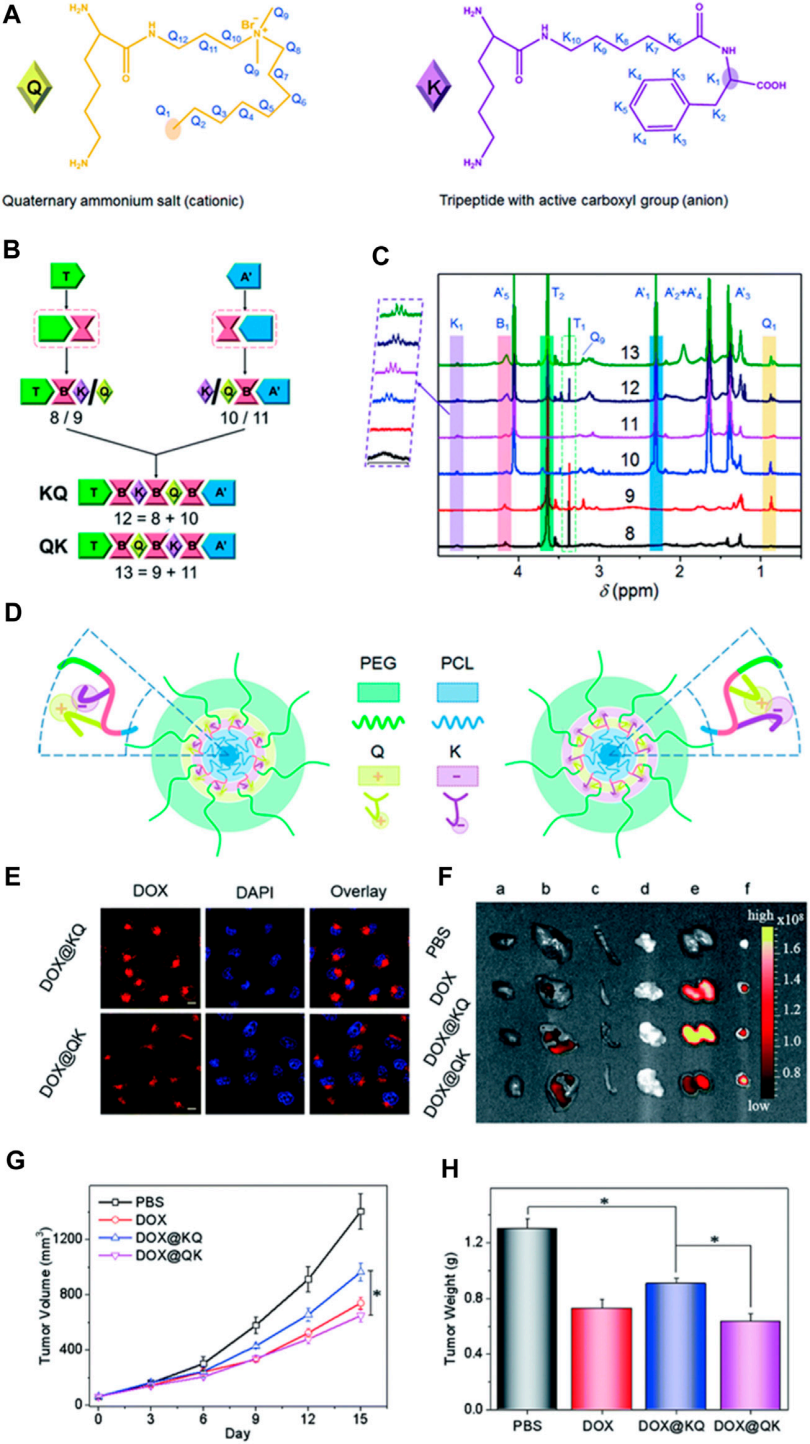
Precise Crafting of Multifunctional Polyurethanes with Clearly Defined Block Sequences. **(A)** Clear structural illustrations of cationic (Q) and anionic (K) functional segments. **(B)** Succinct schematic representations demonstrating the sequence-specific synthesis of the sample. **(C)** Overlay of superimposed 1H NMR spectra for synthetic polyurethanes, showcasing characteristic protons. **(D)** Spontaneous self-assembly of multifunctional polyurethanes with controlled sequences of functional segments; schematic depictions of KQ micelles and QK micelles. Assessment of sequence-controlled multifunctional polyurethanes as carriers for drugs. **(E)** Confocal images of HeLa cells exposed to micelles loaded with DOX for 3 h, with scale bars indicating 10 μm. **(F)** Fluorescence images obtained *ex vivo* from excised organs of nude mice bearing HeLa tumors post intravenous injection with DOX-labeled micelles for 3 h, showing the heart **(A)**, liver **(B)**, spleen **(C)**, lung **(D)**, kidney **(E)**, and tumor **(F)**. Mice treated with PBS served as the control. **(G)** Changes in tumor volume in nude mice following intravenous administration of various drug formulations at a DOX dosage of 2 mg kg^-1^ **(H)** Average tumor weights obtained from mice treated with different DOX formulations over a span of 2 weeks. (Song et al., 2022). Reused under Creative Commons Attribution License.

Producing polyurethanes (PUs) with controlled sequences and block structures remains a significant challenge. Song et al. systematically developed segmented PUs for targeted drug delivery, precisely arranging blocks of cationic and anionic segments. Their research indicates that the organization of these functional segments significantly impacts self-assembly, resulting in unexpected surface charges in the micelles formed. This alteration affects protein absorption, cell uptake, distribution within the body, and the effectiveness against tumors both in laboratory settings and in living organisms (Figure 6). Similarly, Caracciolo et al. utilized amphiphilic PU elastomer networks for drug delivery purposes. These investigations underscore the adaptability of segmental and amphiphilic elastomer network assemblies in creating precise multiblock PUs with intricate structures and diverse functions, thus facilitating the clinical application of PUs in biomedicine (Caracciolo et al., 2012; Song et al., 2022).

A third method involves tailoring the physiochemical properties of PUs. Many biomedical-grade PUs exhibit swelling or degradation in hydrated environments, influenced by factors like hydrophilic segments in PU synthesis. Swelling can impact drug release by altering diffusion path lengths and increasing free volume, affecting drug diffusion rates variably across different drug-polymer systems (Bakhshandeh et al., 2023). Rapid swelling compared to drug release might necessitate modeling drug delivery using swollen state diffusivity and path length. PUs can be designed for degradation or erosion via mechanisms like hydrolysis or oxidation. Here, erosion refers to mass loss, while degradation refers to polymer chain cleavage. Erosion varies between surface and bulk types, impacting drug delivery differently (Park et al., 2007; Liang et al., 2019). Surface erosion reduces PU size, shortening diffusion paths, while bulk erosion can enhance drug diffusivity by loosening the polymer matrix. Medical-grade PUs often experience mixed erosion/degradation methods, potentially impacting drug delivery kinetics depending on the timescale relative to drug release rates. However, the drug release efficiency of the PUs can be enhanced by controlling the degradation of the DDSs. The degradation can be improved by carefully controlling the structure of the DDSs (Kamaci, 2020; Łukaszewska et al., 2023).

## 5 Imaging-guided PUs drug delivery

**FIGURE 7:**
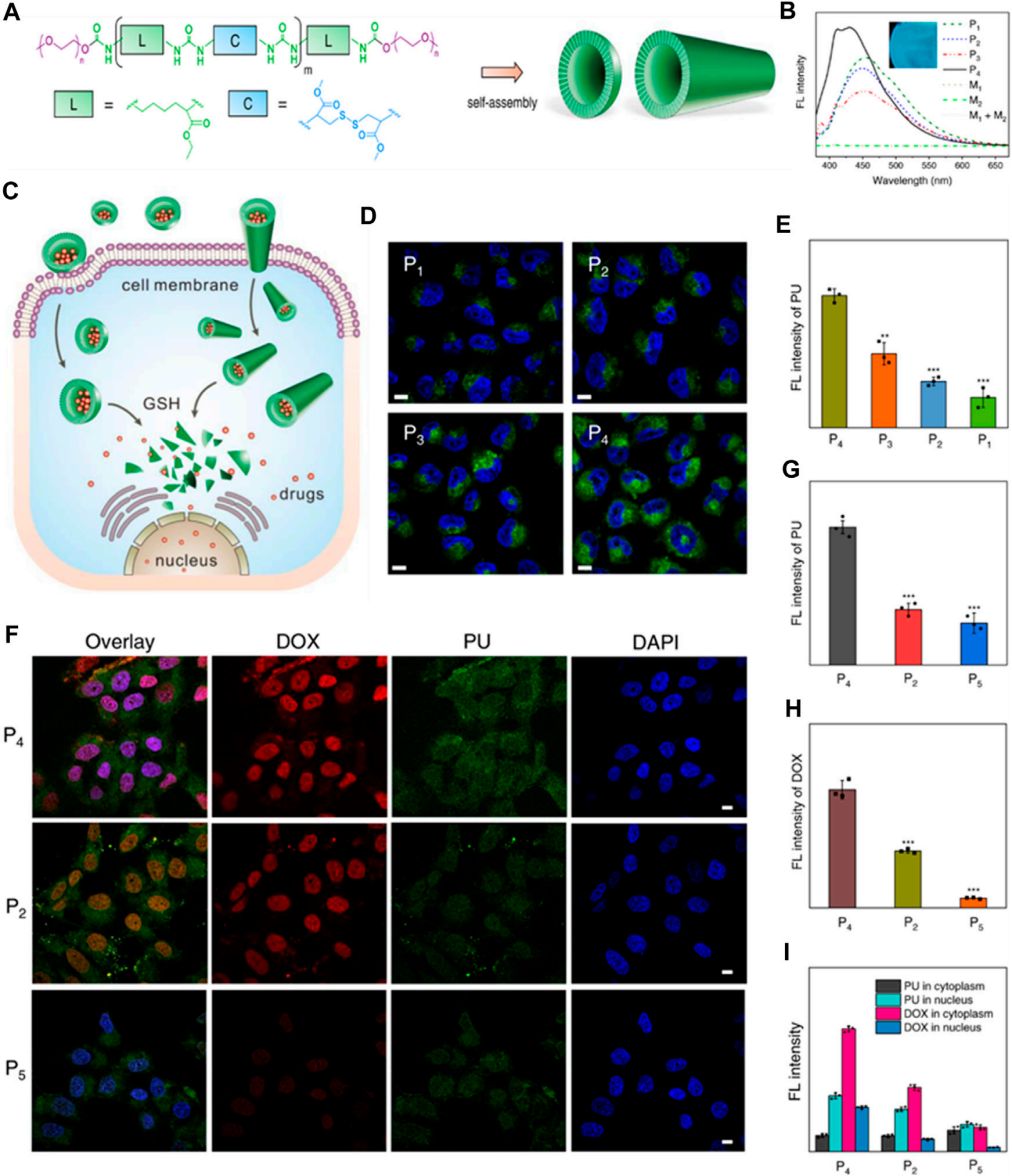
**(A)** Diagram depicting the structure of polyurethanes (PUs) and their arrangements. **(B)** Spectra showing the fluorescence of PU assemblies compared to monomers (1 mg/mL concentration). The inset PU film photograph illuminated by UV lamp of 365 nm. **(C)** Illustration of drug cellular uptake and intracellular delivery properties of PU assemblies with varying conformations and morphologies. **(D)** Confocal laser scanning microscopy (CLSM) images depicting MCF-7 tumor cells treated with the PU assemblies for 2 h. Cell nuclei stained with DAPI (2-(4-amidinophenyl)-6-indolecarbamidine dihydrochloride). The fluorescence from the PU channel appeared green. Scale bars: 5 μm. **(E)** Measurement of fluorescence intensity of PUs in MCF-7 cells after 2 h of incubation. Data presented as mean ± standard deviation (SD). **(F)** CLSM images illustrating MCF-7 tumor cells treated with DOX-loaded PU assemblies for 2 h. Cell nuclei were stained with DAPI (blue). Scale bars: 5 μm **(G, H)** Mean fluorescence intensity of the PUs and DOX, respectively. **(I)** Cytoplasm and nucleus normalized fluorescence intensity (Zhou et al., 2022a). Reused under Creative Commons Attribution license.

Lately, there has been notable attention focused on the advancement of fluorescent polymers due to their inherent benefits, such as minimal toxicity, favorable biocompatibility, extended stability, and improved manufacturability (He et al., 2023; Ma et al., 2023). Specifically, fluorescent polyurethane (PU) stands out as a highly sought-after polymer due to its exceptional blend of distinctive characteristics, including remarkable mechanical durability, effective resistance to abrasion, and superior elasticity. Polyprodrugs have garnered considerable interest as nanomedicines, aiming to enhance drug loading capabilities and minimize toxicity associated with carriers. Polyurethane prodrug nanoparticles and micelles with a stimuli-responsive nature for imaging-guided therapy are gaining momentum as alternatives to non-assimilable zwitterionic materials prepared via living or free radical polymerization. These polymers undergo self-assembly to form vesicles and structures resembling nanotubes, demonstrating size expansion and contraction, metamorphosis, and changes in color mediated by hydrogen bonding. Furthermore, these polymeric structures exhibit extremely high stability and rapid responsiveness, enabling the creation of “on-off” switchable nanocarriers suitable for drug delivery and theranostic applications. Due to their inherent fluorescence, the uptake by cells and the release of drugs inside cells from PU nanoparticles can be conveniently monitored without the need for labeling (Figure 7). (Zhou et al., 2022a)

As an illustration, Qian et al. developed a pH-responsive zwitterionic polyurethane prodrug for improved cancer therapy through drug delivery. The augmented cytotoxicity mechanism of the polymer prodrug micelles was investigated over various durations using both flow cytometry and fluorescence microscopy. Findings revealed that the fluorescence intensity of the prodrug micelles surpassed that of PEG-based micelles at equivalent exposure periods, suggesting a more effective cellular uptake of the prodrug micelles (He Q. et al., 2021).

## 6 Targeted polyurethane drug delivery

Polyurethane-centered drug delivery systems employing targeted distribution mechanisms present an innovative strategy within the pharmaceutical realm. These systems strive to amplify the effectiveness and precision of delivering medications to specific locations in the body, optimizing therapeutic impacts while mitigating potential side effects. Targeted polyurethane drug delivery involves crafting formulations capable of navigating physiological barriers and selectively releasing drugs precisely where intended (Wienen et al., 2023).

**FIGURE 8:**
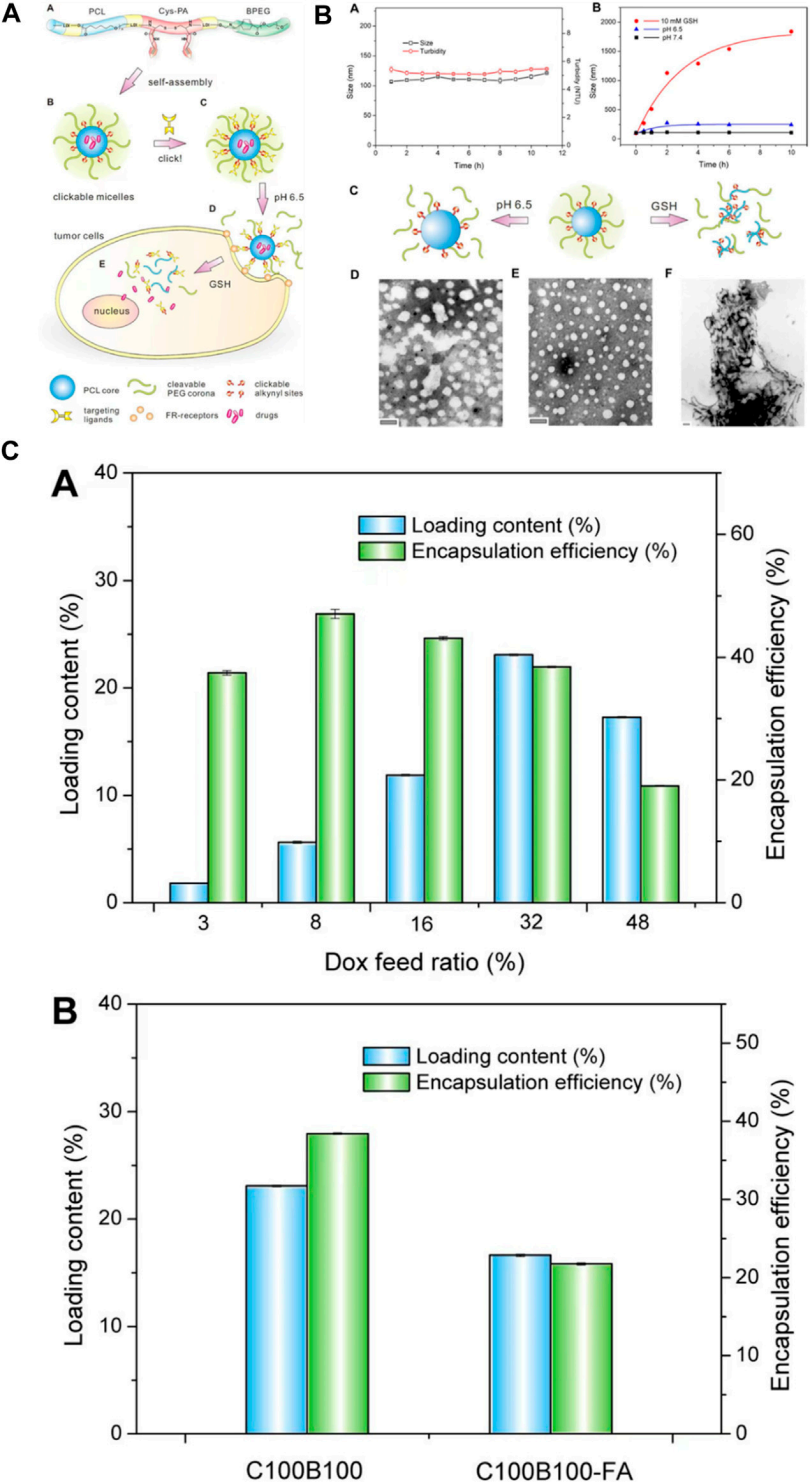
**(A)** The development and formation of targeting-responsive and tumor-degradable PU nanomicelles involves the following: **(A)** illustrating the molecular makeup of multiblock PUs; **(B)** creating self-assembling clickable PU nanomicelles; **(C)** applying click chemistry to attach a folate ligand; **(D)** benzoic-imine linkage cleavage-induced pH-induced PEG shell detachment in the extracellular milieu; and **(E)** releasing drugs intracellularly stimulated by disulfide bond cleavage in response to GSH. **(B) (A)** Alteration in size and cloudiness of polyurethane micelles over time under standard physiological conditions. **(B)** Changes in micellar size within acidic and redox environments. Schematic representations **(C)** and TEM images **(D–F)** of PU micelles before **(E)** and after exposure to pH 6.5 **(D)** and 10 mM GSH **(F)** for 24 h **(C) (A)** Loading capacity and encapsulation efficiency of DOX in clickable and degradable PU micelles at varying drug feed ratios. **(B)** Loading outcomes for different micelles (Song et al., 2013). Reproduced with permission from ref. no. 73. Copyrights 2013, AMERICAN Chemical Society.

Polyurethane carriers can be engineered to transport drugs directly to target tissues or organs, elevating the overall treatment efficacy. By functionalizing polyurethane surfaces, it becomes possible to attach ligands or targeting components that interact with particular receptors at the designated site, refining the accuracy of drug delivery. Additionally, polyurethane drug delivery systems (PU DDSs) can be tailored to respond to specific stimuli like pH, temperature, or enzymatic activity, triggering drug release solely upon encountering the desired conditions. Leveraging polyurethane in nanoparticle form facilitates the creation of carriers boasting high surface areas, enhancing drug loading capacity and enabling precise delivery to specific cells or tissues (Tao et al., 2023). To meet these requirements, Song and colleagues devised an innovative polyurethane nanomicelle with targeting capabilities and tumor-degradable features for the versatile delivery of anticancer drugs. The polyurethane was created by synthesizing biodegradable poly (ε-caprolactone) (PCL) and l-lysine ethyl ester diisocyanate (LDI). It was then elongated using a newly designed l-cystine-derivatized chain extender containing a redox-responsive disulfide bond and clickable alkynyl groups (Cys-PA). The polymer chain terminated with a detachable methoxyl-poly (ethylene glycol) incorporating a benzoic-imine linkage (BPEG) sensitive to pH variations. These resulting polymers demonstrated favorable self-assembly characteristics, responsiveness to stimuli, good compatibility with cells, and a high capacity for loading doxorubicin (DOX). Additionally, folic acid (FA) was effectively attached to the polyurethane micelles as a representative targeting agent using a click reaction. The inclusion of FA resulted in heightened cellular uptake and improved drug efficacy against FA-receptor-positive HeLa cancer cells in laboratory tests. This research serves as a demonstration of a straightforward strategy for designing externally triggered nanocarriers for targeted delivery to tumors and controlled release of drugs within cells (Figure 8). (Song et al., 2013)

Polyurethane-based drug delivery can serve in combined therapies, concurrently delivering multiple drugs to target different aspects of a disease or condition. The release pace of drugs within polyurethane systems can be finely adjusted, allowing for sustained or intermittent drug release as required for optimal therapeutic results. Furthermore, PU carriers can be integrated with imaging agents, enabling real-time tracking of drug dispersion and aiding in evaluating treatment effectiveness (Gajbhiye et al., 2020; Yang et al., 2020).

## 7 Activatable polyurethane drug delivery

Activatable polyurethane drug delivery adheres to the principles of precision medicine, facilitating customized treatments based on individual patient needs and disease characteristics. Incorporating various activation methods or merging activatable polyurethane systems with other therapeutic approaches could offer versatile remedies for addressing intricate medical conditions. Furthermore, activatable systems that blend therapeutic and diagnostic functions (known as theranostics) can provide real-time monitoring of drug release and treatment effectiveness, contributing to more informed healthcare decisions. These systems contribute to the evolution of intelligent drug delivery platforms capable of adapting to changing physiological conditions, thereby improving therapeutic efficacy and minimizing side effects (Zhang and Pu, 2020).

**FIGURE 9:**
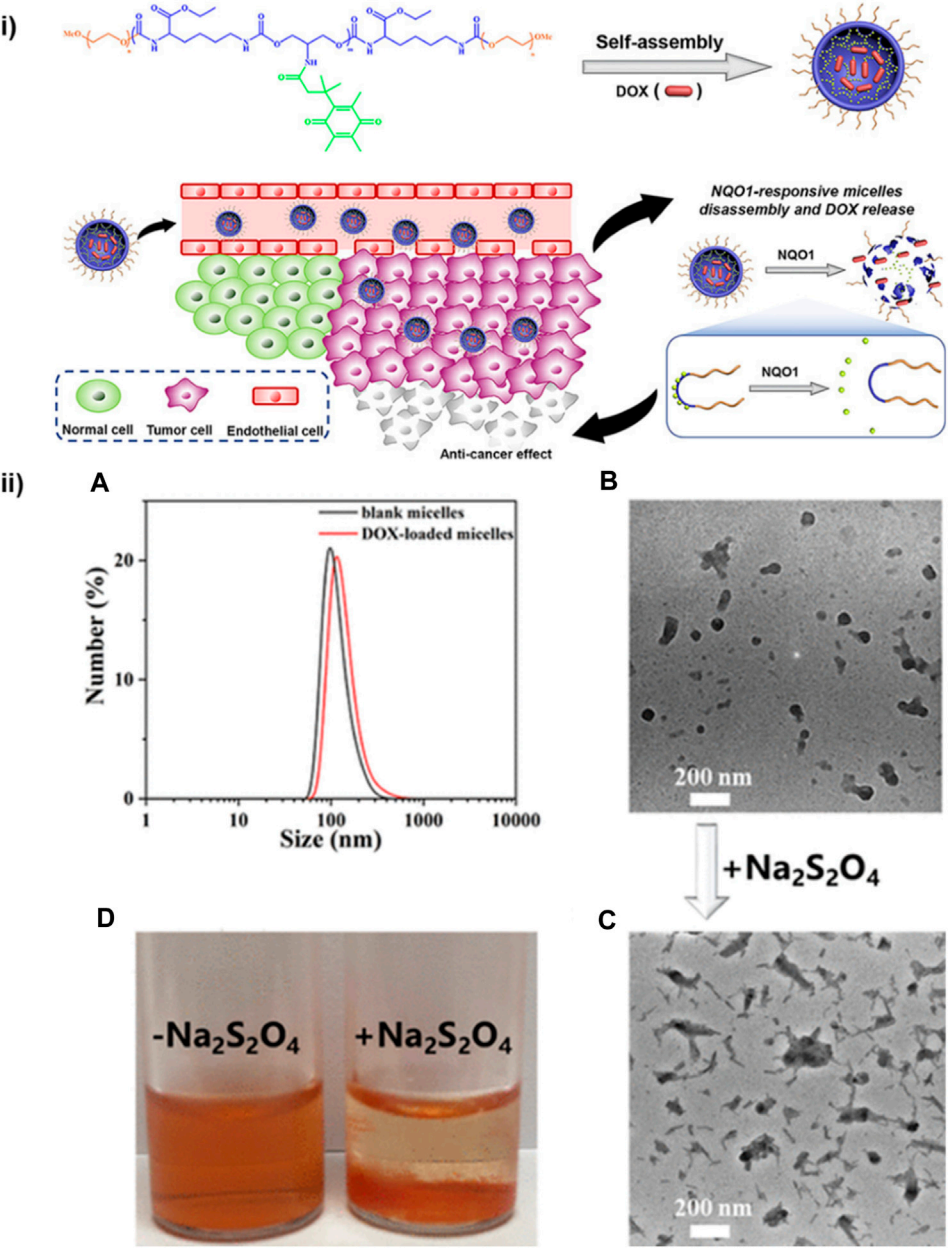
**(i)** PEG-PTU-PEG triblock copolymer micelles responsive to NQO1 for redox-triggered intracellular drug delivery. **(ii**) **(A)** DLS analysis and particle size of PEG-PTU20-PEG blank micelles. **(B)** Drug (DOX) loaded micelles TEM image. **(C)** TEM image and **(D)** digital photograph of drug-loaded micelles after Na_2_S_2_O_4_ induced disassembly (Xie et al., 2023). Reproduced with permission from ref. no. 79. Copyrights 2020, AMERICAN CHEMICAL SOCIETY.

The drug-delivering efficiency of the delivery system can be improved by the integration of a specific stimulus for the drug release. Athar et al. developed redox-responsive cleavable soyabean-coated polyurethane nanomicelles and polyurethane-hyaluronic acid nanomicelles. The prepared drug carriers exhibited controlled self-assembly, stimulus-responsive drug release, excellent biocompatibility and high loading capacity of doxorubicin. All these features are responsible for effective activatable drug delivery by the nanomicelles (Mahdieh et al., 2022). Moreover, for further enhancing the drug delivery efficiency of the polyurethane-based nanomicelles, the material should be amphiphilic. Yu et al. synthesized amphiphilic micelles of polyurethane combined with methacrylate polyethylene glycol (mPEG) by sulphur linkages through ring opening reaction used for hydrophobic anticancer drug delivery (Yu et al., 2020). Jinhai Xie et al. have designed diol monomers based on trimethyl-locked benzoquinone (TMBQ) amphiphilic PU (PEG-PTU-PEG). The synthesized A-B-A-type, enzyme-responsive functional block copolymer PEG-PTU-PEG successfully encapsulates doxorubicin (DOX) in nanomicelles through self-assembly (Figure 9). Drug release and micelle disintegration result from the NAD(P) H: quinone oxidoreductase 1 (NQO1) enzyme eliminating TMBQ by lactonization through two-electron reduction, upsetting the hydrophilic–hydrophobic equilibrium of the PU micelle structure (Xie et al., 2023).

**FIGURE 10:**
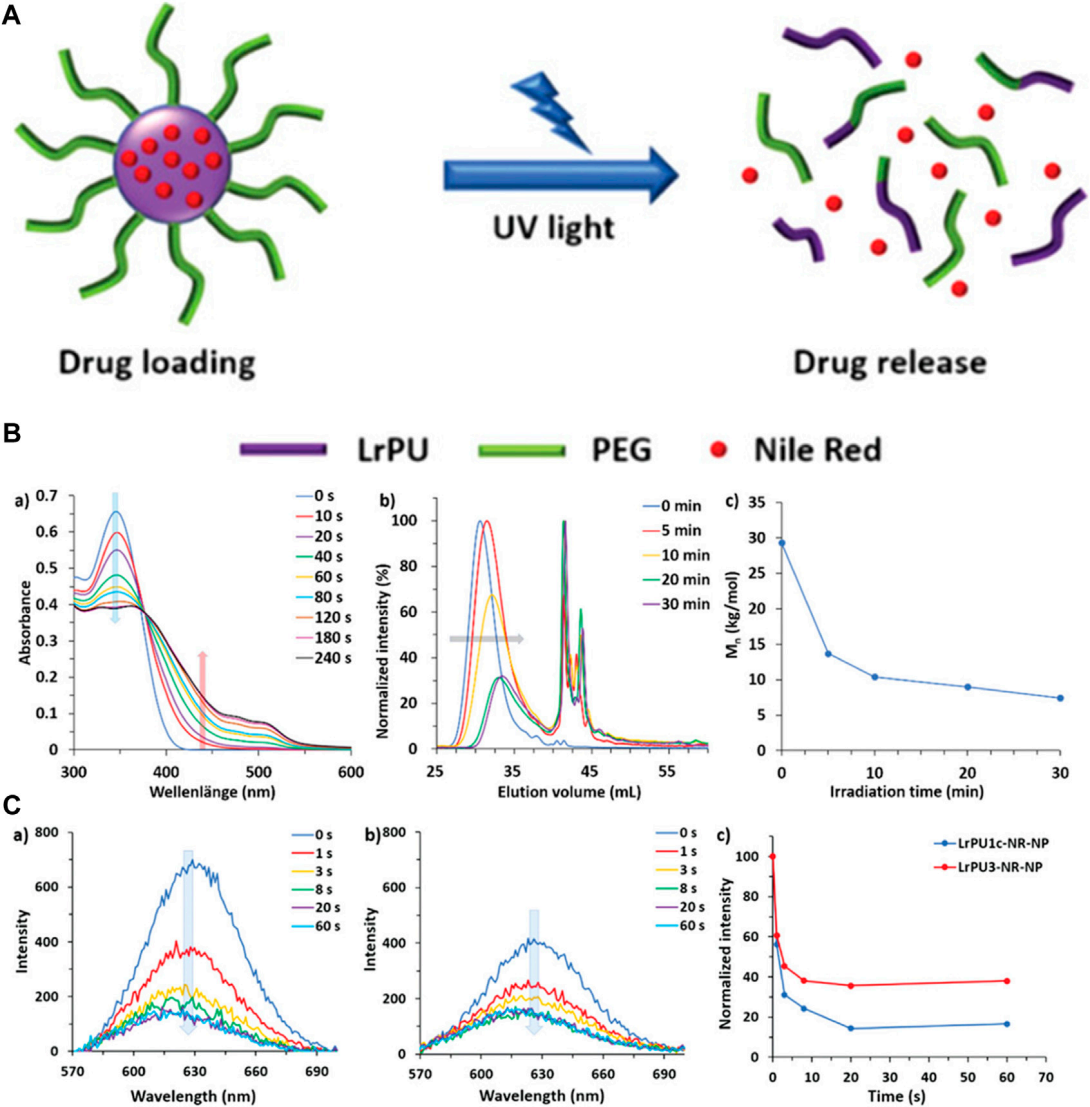
**(A)** Diagrammatic representation illustrating the creation and structural evolution of LrPU nanoparticles containing Nile Red as a representative drug model. **(B)** Changes observed in **(A)** UV–visible absorption spectra, **(B)** SEC (Size Exclusion Chromatography) profiles, and **(C)** Mn (number-average molecular weight) of LrPU2a after exposure to UV light. **(C)** Emission spectra of Nile Red-loaded **(A)** LrPU1c-NR-NP and **(B)** LrPU3-NR-NP nanoparticle solutions when subjected to UV light for varying durations; **(C)** diagrams demonstrating the light-responsive release of Nile Red trapped within the nanoparticles (Sun et al., 2019). Reused under Creative Commons Attribution License.

The macromolecular drug delivery agents are not so efficient in drug delivery and are not so responsive to a specific stimulus. To solve this problem, Chen et al. synthesized unimolecular micelles for the drug delivery approach instead of supramolecular micelles. He utilized dendritic polymer micelles with a polar framework for chemotherapeutic drug delivery, which has proved to be more effective in pH-responsive drug delivery due to the protonation of the dendritic polymer. The efficiency of the drug delivery agent has been improved due to the simplified chemistry of the urethane. The unimolecular micelles have also been employed for controlled drug release during chemotherapy (Chen and Liu, 2022). The internal bodily stimuli are proved to be not so effective in the case of controlled drug delivery. To enhance the sensitivity of the controlled delivery system, Sun et al. developed a monodispersed light-responsive polyurethane delivery system. The monodispersed nanoparticles loaded with the drug undergo rapid degradation upon light irradiation. The degradation of the delivery system has been investigated by the DLS method. The monodispersed nanoparticles loaded with a model drug Nile Red when irradiated with UV light degraded rapidly releasing the drug that was detected by fluorescence spectroscopy (Figure 10). This light-responsive polyurethane drug delivery can be the most advanced drug delivery approach (Sun et al., 2019).

Zhou and colleagues investigated the efficacy of delivering polyurethane-polyurea nanocapsules (Puua-NCs) with a shell that undergoes pH-induced cationization and redox-triggered release to the vascular system. They integrated the fluorescent lipophilic dye DiI into Puua-NCs of various sizes and concentrations. Cellular uptake studies conducted on human aortic endothelial cells revealed a dose-dependent uptake of these Puua-NCs. In a mouse model of atherosclerosis, particularly apolipoprotein E-deficient mice fed a Western diet, the circulating Puua-NCs remained stable and accumulated in the aortic endothelium and lesions within 24 h post intravenous administration. Treatment with thiol-reducing and oxidizing agents disrupted the disulfide bonds on the surface of internalized nanocapsules, leading to their disassembly and the release of the encapsulated cargo within cells. Ultimately, the research suggests that Puua-NCs have the potential to function as a redox-controllable drug delivery system for cardiovascular purposes (Zhou et al., 2022b).

Continuous advancements in nanotechnology are pivotal in refining activatable polyurethane systems, enabling the creation of smaller, more efficient carriers with enhanced drug-loading capabilities. Activatable drug delivery systems hold promise for tackling diseases typically challenging to treat due to drug resistance or complex underlying conditions. Overcoming these hurdles and exploring these perspectives will be critical in advancing activatable polyurethane drug delivery and translating it into practical, impactful clinical applications. Collaborative endeavors among researchers, clinicians, and industry partners will be instrumental in surmounting obstacles and harnessing the full potential of this technology (Bil et al., 2020; Cheng et al., 2021).

## 8 AI and machine learning in polyurethane drug delivery

AI algorithms streamline the analysis of extensive datasets concerning polyurethane properties, drug interactions, and patient reactions. This optimization process significantly expedites the creation of specialized drug delivery systems. By employing machine learning models (MLMs), it becomes possible to forecast how polyurethane drug delivery systems will behave within a living organism. This encompasses foreseeing drug release rates, spotting potential side effects, and enhancing the system’s design for better therapeutic results (Suwardi et al., 2022).

Furthermore, AI plays a pivotal role in tailoring drug delivery systems to individual patients based on their unique genetic makeup, metabolic processes, and disease profiles. This shift towards personalized medicine has the potential to enhance treatment effectiveness while minimizing adverse effects. ML algorithms can be utilized to develop intelligent polyurethane systems that adjust drug release rates in response to real-time changes in the body, thereby enhancing precision and efficacy in drug delivery (Pugar et al., 2022).

The contribution of AI extends to swiftly evaluating the compatibility of polyurethane materials by predicting immune responses or toxic reactions. This accelerates safety assessments and ensures the reliability of drug delivery systems. By amalgamating data from various sources such as molecular databases, clinical trials, and patient records, AI systems offer comprehensive decision-making support to researchers and healthcare professionals engaged in polyurethane drug delivery (McDonald et al., 2023).

AI-powered algorithms also automate the optimization of designs for polyurethane drug delivery systems, efficiently exploring a vast array of possibilities that might not be discernible through conventional methods. Real-time monitoring of patient responses and system performance is made feasible through AI, enabling machine learning models to analyze this data and prompt adaptive changes in drug delivery parameters for ongoing optimization during treatment. AI algorithms can identify patterns associated with drug resistance, aiding in the proactive design of polyurethane drug delivery systems to counteract or mitigate resistance mechanisms (Sagdic et al., 2022).

**FIGURE 11:**
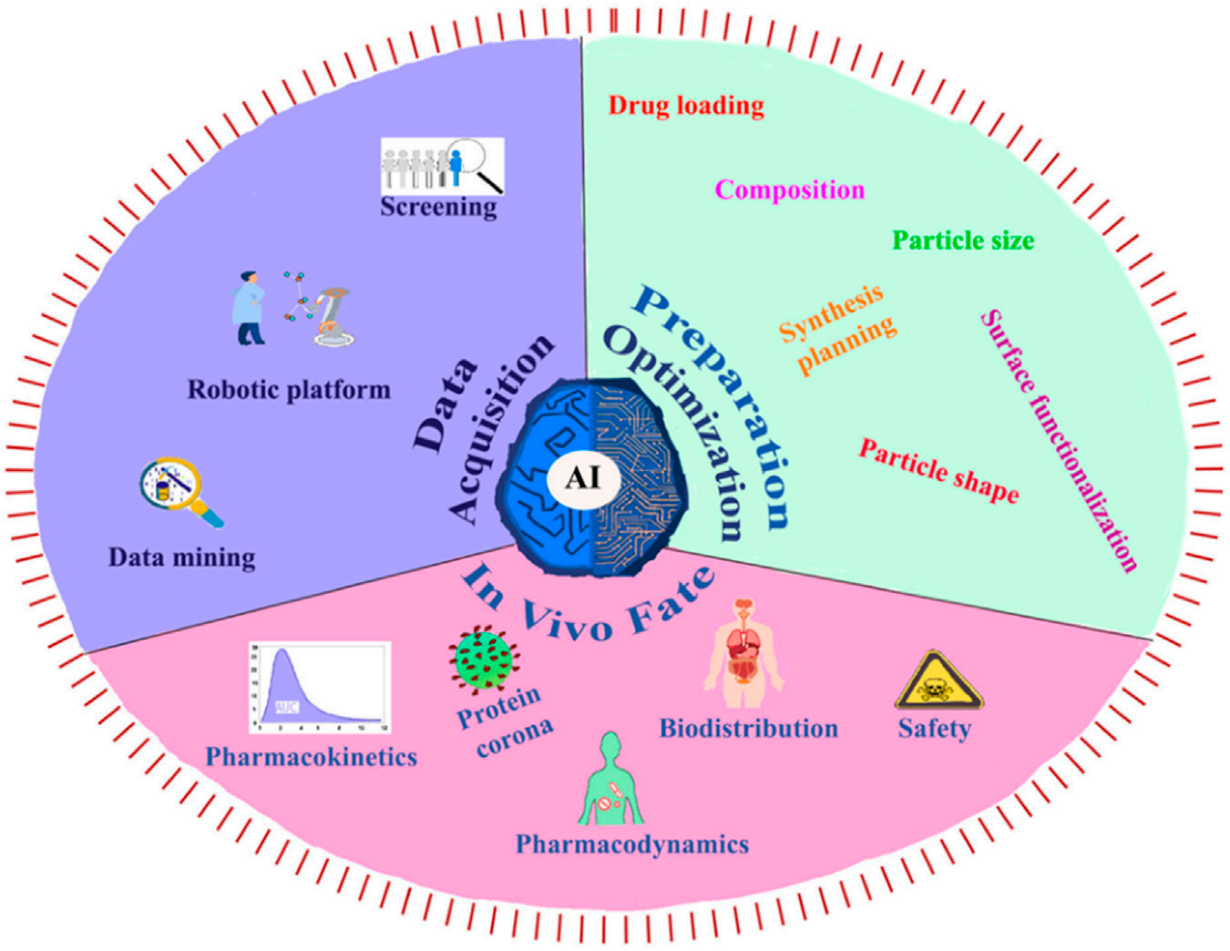
**(A)** The present exploration and utilization of AI in the context of data acquisition, preparation optimization, and *in vivo* fate (Mozafari et al., 2023). Reproduced with permission from ref. no. 90. Copyrights 2023, american chemical society.

The integration of AI and ML into polyurethane drug delivery systems holds immense potential for advancing precision medicine, expediting drug development, and ultimately enhancing patient outcomes. This advancement necessitates collaboration across disciplines, robust validation procedures, and continuous regulatory oversight to ensure the responsible and ethical application of these technologies in healthcare (Zohuri and Behgounia, 2023). Utilizing AI in nanomedicine in a number of areas, including data collection, nanosystem setup and optimization (including physicochemical, pharmacological, and synthetic parameters), and the fate of nanomedicine *in vivo* **(** Figure 11). (Mozafari et al., 2023)

The integration of AI and ML into PU DDSs represents a groundbreaking advancement in the field of pharmaceutical technology. These intelligent systems leverage AI algorithms to optimize drug release profiles, enhance targeting precision, and personalize therapeutic regimens. ML models analyze vast datasets to predict patient responses, enabling tailored drug delivery strategies that consider individual variations in metabolism and physiology. Additionally, AI algorithms can optimize the design and fabrication of polyurethane-based drug delivery platforms, ensuring efficient and controlled release kinetics. This integration not only improves the overall efficacy of drug delivery systems but also opens avenues for real-time monitoring and adaptive adjustments, thereby revolutionizing the way therapeutic agents are administered. The synergy between AI, ML, and PU DDSs holds immense promise for advancing precision medicine and improving patient outcomes in the realm of pharmaceuticals (He S. et al., 2021).

## 9 Challenges and future perspectives

PU nanoparticles are emerging as the most advanced drug delivery agents in the medical realm. The efficiency and functionality of the PUs DDSs can further be enhanced by carefully integrating modern features and by controlling their structure and design. PUs DDSs can act as state-of-the-art drug delivery agents, but a lot of research and practical work is needed to bring PUs from laboratories to hospitals. The controlling design and structure of PUs for targeted drug delivery and activatable drug delivery is a very challenging task to be accomplished. AI and Machine Learning can also be used to design modern PU DDSs for different pathological conditions. If proper research work is done to meet all these projected features, PU DDSs can act as the most effective delivery system in the biomedical field.

## 10 Conclusion

Polyurethanes (PUs) are intriguing polymers with diverse properties that make them highly suitable for applications in drug delivery and various biomedical contexts. These applications encompass nanoparticulate systems, stimuli-responsive scaffolds, and imaging-guided therapy. While PUs are extensively investigated for diverse biomedical uses, it is imperative to evaluate their actual impact on the healthcare system critically. The pharmaceutical sector is experiencing a rapid surge in the development of PU-based drug delivery systems for treating different medical conditions. A novel approach to designing PU-based medication delivery systems is emerging, facilitated by the integration of perspectives from both synthetic and biological sciences.

The distinctive characteristics of materials based on PUs present new possibilities for addressing significant challenges in drug formulation and developing advanced drug delivery systems with innovative capabilities. Overcoming the issue of poor drug solubility in PU formulation involves depositing large amounts of drugs in the amorphous state onto the PU nanoparticles skeleton. The extensive surface area, coupled with compatibility with different solvent systems, facilitates interaction with the aqueous environment and promotes the diffusion of dissolved drugs. Water uptake performance of PU nanoparticulate systems in various pH media reveal that these exhibit a higher swelling capacity in acidic environments compared to neutral and alkaline media. Biodegradation experiments involving enzymatic and hydrolytic degradation with amino acids or biodegradable components indicate a higher biodegradation rate.

Alternatively, the release patterns of drugs from PU-based materials can be controlled using various methods, such as combining swellable/erodible skeleton components, adjusting the intensity of drug/skeleton interactions, coating with membrane-like substances, and incorporating stimuli-responsive components.

Current manufacturing protocols for PU materials allow for the utilization of nearly any organic or inorganic material as a skeleton component, adaptable to formulate drugs with diverse physicochemical features and stability. However, additional *in vivo* studies are necessary to address safety concerns related to the biomedical applications of PU materials. While existing literature on PU materials for drug delivery primarily focuses on bench-scale production, the growing interest in their pharmaceutical applications encourages experimentation on a pilot scale as an initial step toward industrial production. Subsequent efforts should concentrate on transforming drug-loaded PU-based materials into practical medicines, exploring possibilities such as tablets, capsules, ointments, inhalation powders, or injectable formulations. It is crucial to retain the unique properties of PU materials during this transformation process.
